# Some musings on our labor's meaning and the legacy of our departing friends; on who, why, and how we should publish obituaries

**DOI:** 10.1120/jacmp.v17i4.6541

**Published:** 2016-07-08

“Life is pleasant. Death is peaceful. It's the transition that's troublesome.”

— Isaac Asimov

“Now he has departed from this strange world a little ahead of me. That signifies nothing. For us believing physicists the distinction between past, present, and future is only a stubbornly persistent illusion.”

— Albert Einstein

“When some men die it is as if you had lost your pen‐knife, and were subject to perpetual inconvenience until you could get another. Other men's going is like the vanishing of a great mountain from the landscape, and the outlook of life is changed forever.”

— Phillips Brooks

For the living, the ultimate entropy is death. The human struggle, therefore, is both against death and also against all incremental steps in the journey toward the inevitable. At bottom, all vocations involve a struggle against entropy and a fight for a better life, an easier life, a longer life, and a higher quality of life. The practice of medicine is such a vocation, confronting the ultimate entropy with its devotion to saving of life, preservation of live, improvement in quality adjusted life‐years, and hospice care when the inescapable arrives.

It is said, with tongue in cheek, that the goal of specialty medicine is for the specialist to assure that the patient dies consequent to a disease process of someone else's specialty. However, the specialty practice of medical physics is mostly exempt from this waggish observation. No one (at least I hope almost no one) blames the medical physicist when the grim reaper arrives. Most patients have experienced a radiation‐based imaging or therapy procedure somewhere along their journey. When the time comes, our practice historically is mostly off the hook; the flip side of this is that our contribution may be overlooked and forgotten.

That does not mean that the practice of the medical physics specialty is any less significant, any less essential to resisting the walk of the final mile. Proper imaging leads to proper diagnosis, which leads to proper treatment. Better definition of targets, more specific and conformal deliver of dose, better fractionation, and better biological response define proper treatment. Discovery and development of new imaging and treatment technologies, along with responsible clinical practice, define the dignity of our profession.

So when one of our own passes, we experience a double tragedy. We lose a lifetime of knowledge and experience, of loyalty and humor, of equity and virtue. In addition, we lose that individual's service in the struggle against entropy, and thousands or millions of patients have lost a friend who would stand with them. I believe this is true of every practicing medical physicist.

This year, I lost two friends who were instrumental in the founding of the *JACMP*. Ann Wright, who first suggested that clinical physicists need a standalone journal, and Alex Turner, who chaired the ACMP the year the process to create the journal was initiated. Ann and Alex had many friends; we will miss them greatly.

Now let us discuss some process and procedures. Ann's obituary will be published in *Medical Physics*, and we are preparing an obituary for Alex to be published in the *JACMP*. So how are these decisions made? Whose obituary is published where and who qualifies? On the flip side, who is left out? It is obviously very desirable to have these questions answered in advance, to avoid offence and to properly honor our colleagues.

The AAPM has a policy on how to address the Honoring of Recently Deceased Members: http://www.aapm.org/org/policies/details.asp?id=369&type=AP.

Briefly, the policy defines those worthy of special recognition as members who have served the AAPM in one of the following capacities: AAPM President, Coolidge Award Winner, Journal Editor‐in‐Chief, Nobel Laureate.

These are some pretty lofty achievements and not many of our members meet these criteria. The policy is somewhat flexible and does allow for others to be recognized upon the recommendation of the Awards and Honors Committee and approval of the Board of Directors. Both *Medical Physics* and *JACMP* editors control the policy of who is recognized in a memorial. Although not specifically mentioned in the AAPM policy, the editors of *Medical Physics* have generally followed this guideline in identifying those who will have memorials published in its pages, although occasionally others are recognized.

So I am proposing the following: The *JACMP* will plan to publish memorials for those in the following categories who do not otherwise meet the AAPM policy criteria (and therefore do not qualify for an obituary in *Medical Physics*): ACMP Chair, Williams Professional Achievement Award Winner, Quimby Lifetime Achievement Award Winner, *JACMP* Editor‐in‐Chief.

Others may also be selected, based on the editors' decision. Now for the why: Ann Wright qualifies as a former AAPM President under the AAPM policy and that is why her memorial will be published in *Medical Physics*. Alex Turner qualifies as an ACMP Chair and his obituary is in preparation for the *JACMP*. This seems to be a reasonable solution to honor significant individuals who do not meet the AAPM policy criteria.

Still, this overlooks a lot of people. I am talking about those who get up at 3:00 a.m. in order to pull a brachytherapy implant on time. I am talking about those who reserve the beam scanner between midnight and 6:00 a.m. Monday morning because that is the only time it is available. I am talking about those who use their gentle powers of persuasion to argue with a radiologist that 22 kV is not an appropriate energy for mammography. In short, I am talking about those who provided better care for their patients at significant personal sacrifice. We need to honor the medical physicist in the trenches. It is regrettable that it is not practical to publish an obituary in the *JACMP* for every physicist who passes. We do not have the space or the volunteer labor to do this.

As of 2016, only 115 total Coolidge, Williams, and Quimby awards have ever been given. With duplicates, only 96 individuals have ever won any of these awards. There have been 8 Editors‐in‐Chief of *Medical Physics* and 4 Editors‐in‐Chief of the *JACMP*. There are 58 AAPM Presidents and 28 ACMP Chairpersons. Again with duplications, since 1958 (the founding of the AAPM), only about 150 AAPM members meet one or more of these criteria for recognition in either journal, out of 15,000 or so members over the years. Truly, we have selected those whose “going is like the vanishing of a great mountain from the landscape, and the outlook of life is changed forever”.

I want to thank Timothy Solberg and Per Halvorsen, Associate Editors‐in‐Chief of the *JACMP*, for their valuable and perceptive comments.

## COPYRIGHT

This work is licensed under a Creative Commons Attribution 3.0 Unported License.

Michael D. Mills, PhD

Editor‐in‐Chief, JACMP

